# Serum Vitamin D Level Does not Affect The Sensitivity of Parathyroid Adenoma Localization Tests

**DOI:** 10.1038/s41598-019-48536-z

**Published:** 2019-08-19

**Authors:** Muhammed Erkam Sencar, Davut Sakiz, Ilknur Ozturk Unsal, Sema Hepsen, Murat Calapkulu, Pelin Gumus, Bekir Ucan, Mustafa Ozbek, Erman Cakal

**Affiliations:** 1 Department of Endocrinology and Metabolism, University of Health Sciences, Diskapi Yildirim Beyazit Training and Research Hospital, Ankara, Turkey; 2 Department of Internal Medicine, University of Health Sciences, Diskapi Yildirim Beyazit Training and Research Hospital, Ankara, Turkey

**Keywords:** Parathyroid diseases, Endocrine system and metabolic diseases

## Abstract

The aim of the present study was to evaluate the predictive value of 25-hydroxyvitamin D, calcium and parathormone level, co-existence of thyroid nodule, thyroidectomy history and adenoma volume on the success of neck ultrasound (US) and technetium-99m sestamibi (MIBI) scan in primary hyperparathyroidism (PHP) patients. This study included 256 patients with PHP who underwent parathyroidectomy. 169 (%66) patients had vitamin D deficiency and 56 (%22) of patients had insufficiency. The sensitivity of US and MIBI studies showed no difference between vitamin D deficiency, insufficiency and replete groups (%80.5, %82 and %71 (p > 0.05) and %81, %84 and %71 respectively (p > 0.05)). Vitamin D level was not found to be an independent predictor of localization on either US or MIBI scan after adjusting for different variables (p > 0.05). Calcium level was found to be an independent predictor for US sensitivity (r^2^:0,033, p:0,032) and parathormone level for MIBI sensitivity (r^2^:0,05, p:0,025). The co-existence of nodular thyroid disease and history of thyroidectomy significantly decreased the sensitivity of US (%76 and %43). MIBI sensitivity was not impaired by nodular disease but the history of thyroidectomy also impaired the sensitivity of MIBI (%43). As a result vitamin D level does not affect the sensitivity of preoperative localization tests.

## Introduction

Primary hyperparathyroidism (PHP) is a common endocrine disorder caused by overactivation of one or more parathyroid glands resulting in inappropriate secretion of parathyroid hormone (PTH) by calcium (Ca) level. Surgery provides the only curative treatment for PHP. Lately mini-invasive parathyroidectomy (MIP) has become increasingly common, with equivalent cure and lower complication rates compared with traditional bilateral neck exploration^1^. Accurate localization of parathyroid adenoma is required for MIP. Ultrasonography (US) and MIBI scan studies are the first choice and the most widely used imaging techniques for evaluating the localization of parathyroid adenoma. There are studies in the literature that have found that some preoperative factors such as Ca level, comorbid thyroid disease and multiple parathyroid gland disease may affect the sensitivity of preoperative imaging studies^2^. These factors can predict the results of localization studies and may be effective in the selection of the initial test^3^. Vitamin D deficiency (VDD) is common in PHP and is associated with a more severe form of the disease^4,5^, and PHP patients with VDD present with adenoma of a larger size^6,7^. A report of an large series of PHP patients showed that those with lower 25-hydroxyvitamin D (25(OH)D) levels presented with more severe disease, larger gland volumes and more positive MIBI scans results^8^. It can also be expected that factors affecting vitamin D levels may also affect the disease severity or success of localization studies. Changes in PTH level and disease severity due to seasonal vitamin D variations have been shown in several studies^9–11^. Although there are many studies about the factors affecting the sensitivity of localization studies in the literature, there are few studies with conflicting results, which have investigated the effect of vitamin D level on the success of localization studies.

The study aimed to investigate the effect of the preoperative factors such as vitamin D, Ca and PTH level, co-existence of thyroid nodule, thyroidectomy history, parathyroid adenoma volume and weight on the success of localization studies in PHP patients.

## Material and Method

### Patients

This was a single-center, retrospective trial, compiled from the database of patients diagnosed with PHP who underwent parathyroidectomy between January 2015 and June 2018 at our institution. Approval for this retrospective study was granted by the University of Health Sciences Diskapi Yildirim Beyazit Training and Research Hospital Ethics Committee and informed consent was signed by enrolled patients preoperatively. This study was conducted in accordance with the principles stated in the Helsinki Declaration. All those included in the study were assayed for 25(OH)D, total Ca, and Parathormone (PTH) levels and underwent both parathyroid US and MIBI scan studies preoperatively. Patients were excluded if they had been prescribed calcium and vitamin D supplements during the 3 months before diagnosis, or if they were diagnosed with secondary or tertiary hyperparathyroidism.

The diagnosis of PHP was established by the presence of hypercalcemia and concomitant inappropriately high serum PTH levels or a normal calcium level and high serum PTH levels (normocalcemic hyperparathyroidism) on at least two separate tests with an indication of PHP; low bone mineral density, hypercalciuria, recurrent kidney stone and imaging finding. All patients with symptomatic PHP and asymptomatic patients with indications for surgery according to the 4th International Guidelines for the Management of Asymptomatic PHPT^12^ underwent MIP or bilateral exploration. A bilateral approach was preferred in cases requiring thyroidectomy or for patients with discordant or unsuccessful preoperative localization studies. In surgical failure patients a third localization study were performed before second surgery.

### Method

Serum Ca levels were assayed using a Beckman Coulter Olympus AU5800 Clinical Chemistry autoanalyzer (Beckman Coulter Inc., Brea, California, USA). Serum 25(OH)D and PTH were measured using a Beckman Coulter UniCel DxI 800 immunoassay systems autoanalyzer (Beckman Coulter Inc., Brea, California, USA). Reference ranges were defined as calcium: 8.7–10.4 mg/dl; PTH: 19.8–74.9 pg/ml and 25(OH)D level: 10–42 ng/ml respectively. According to serum 25(OH)D levels, patients were divided into three groups; ≤20 ng/mL (vitamin D deficient), >20 to <30 ng/mL (vitamin D insufficient) and ≥30 ng/mL (vitamin D replete)^13^. Thyroid and parathyroid US was performed by the authors using Hitachi HI Vision Prerius (Hitachi, Tokyo, Japan), a linear 13 MHz probe. All scintigraphic images; early and late static planar images of the skull, neck and upper thorax and also spect images were acquired using a dual-head gamma camera (Siemens ecam-signature; Siemens, Hoffmann Estates, Illinois, USA). Adenoma weight, operative notes, pathology reports, and sestamibi scan results and drug prescriptions were all reviewed from the database. The volume of parathyroid adenoma was calculated using the ellipsoid volume formula (ml) (Length (cm) x Width (cm) × Thickness (cm) × π × 4/3).

### Statistical analysis

Statistical analyses were performed using SPSS software (version 21.0, SPSS, Chicago, IL). Categorical data were summarized with frequencies and percentages (%). All continuous variables with normal distribution were expressed as mean ± standard deviation (SD) and non-normally distributed variables were expressed as median (range) values. The Independent Samples t-test was used to compare continuous variables with normal distribution and the Mann-Whitney *U* test was used for non-normally distributed variables. The sensitivity of localization studies was compared with the Pearson Chi-Square test. Logistic regression analysis was performed to assess the relationship between pre-operative variables (age, gender, serum calcium and PTH level, concomitant thyroid disease, history of operation) and localization study outcomes. Correlations were analyzed using Spearman’s correlation analyses for non-parametric variables and Pearson correlation analysis for parametric variables. Kruskal-Wallis test was used for compare numeric non-normally distributed variables and Anova test was used for compare numeric normally distributed variables. A value of p < 0.05 was considered statistically significant.

### Ethical approval

The study was approved by the Ethics Committee of our institute.

### Informed consent

All participants were informed about the research protocol and all declared voluntary participation with assigned written assent.

## Results

A total of 256 patients who underwent parathyroidectomy for treatment of PHP were included in the study, comprising 214 (%84) females and 42 (%16) males with a mean age of 56 ± 12 years. Full demographic and clinical data are reported in Table 1. 164 (%64) patients had at least one thyroid nodule and 85 (%33) patients had no nodular thyroid disease. 7 (%2.7) patients had thyroid surgery before the diagnosis of PHP. Consistent with the diagnosis of PHP, median serum Ca and PTH levels were high (11 mg/dl (9.3–17), 134 pg/ml (28–1182) respectively) in PHP patients.

**Table 1:** Demographic and clinical data of PHP patients.

	Whole Group (%)	Vitamin D			
		≤20 ng/ml (%)	20–30 ng/ml (%)	≥30 ng/ml	p
Patients (n)	256 (%100)	169 (%66)	56 (%22)	31 (%12)	—
Female/Male	214/42	138/31	48/8	28/3	—
Age (years)	56 ± 12	57 ± 12	56 ± 10	56 ± 13	0.907
Patients with thyroid nodules	164 (%64)	111 (%65)	37 (%66)	16 (%52)	0.300
Ca (mg/dl)	11.1 (9.3–17)	11.1 (9.7–17)	11 (9.3–12.7)	11 (9.5–12.3)	0.139
PTH (pg/ml)	134 (28–1182)	166 (28–1182)	118 (45–431)	110 (63–271)	**<0.001**
25(OH) D (ng/ml)	16.1 (3–56)	11 (3–20)	23 (20.3–29)	38 (30–56)	**<0.001**
Adenoma weight (gr)	0.9 (0.1–45)	1 (0.1–45)	0.70 (0.13–5)	0.56 (0.14–5)	0.078
Adenoma volume (cm^3^)	4 (0.2–293)	5 (0.2–293)	2.4 (0.2–17)	2.5 (0.25–14)	**0.019**

Variables are not normally distributed are presented as Median (range) and other variables with a normal distribution are represented as mean ± SD. Ca: Calcium; PTH: Parathyroid hormone; 25(OH)D: 25-Hydroxyvitamin D.

Vitamin D deficiency was present among %66 of patients, whereas %22 were vitamin D insufficient and %12 were vitamin D sufficient (Table 1). Most of the patients were diagnosed in winter, while the least patients were diagnosed in summer (%32 vs %18 respectively). As expected, the lowest 25(OH)D level was detected in winter, while the highest level of 25(OH)D was detected in summer (17 ± 11 ng/ml vs. 21 ± 10 ng/ml, respectively p:0.449). In the analyses no seasonal variation was detected in 25 (OH) D levels of the participants (p:0.071). A high prevalence of VDD was found among subjects included in the study; 169 of 256 patients (%66) had vitamin D deficiency and 56 (%22) patients had vitamin D insufficiency and 31 (%12) patients with replete vitamin D. The median plasma level of 25(OH)D for the entire study group was 16.1 ng/mL (range, 3–56 ng/mL). The median serum 25(OH)D levels in the deficient, insufficient and sufficient group were 11 (3–20), 23 (20,3–29) and 38 (30–56) ng/mL, respectively. Serum Ca level was not statistically different between 3 groups (11.1 (9.7–17) vs. 11(9.3–12.7) vs. 11 (9.5–12.3) mg/dl, p:0.139) but PTH level was higher in VDD group compared to the vitamin D insufficient and sufficient group (166 (28–1182) vs. 118 (45–431) vs. 110 (63–271) pg/ml p < 0.001) (Table 1). Postoperative histopathological examination demonstrated adenoma in 228 (%89) cases, hyperplasia in 20 (%7.8) cases, carcinoma in 3 (%1.2) cases and pathological parathyroid glands were not detected in 5 (%2) cases, which were surgical failures (Table 2). Parathyroid adenoma weight was not different between 3 groups (p:0.078) but volumes were significantly higher in vitamin D deficient group compared to the vitamin D insufficient and sufficient group (5 (0.2–293) vs. 2.4 [0.2–17] vs. 2.5 (0.25–14) mL, p:0.019) (Table 1). In the correlation analysis, no relationship was detected between Vitamin D level and serum Ca level (p > 0.05) but there was an inverse correlation between serum 25(OH)D level and serum PTH concentration (p < 0.001). There was an inverse correlation between Vitamin D level and resected gland weight (p:0.026) and volume (p:0.009).

**Table 2:** Pathological features.

Pathological features	Patients (n = 256)
Single gland adenoma	225 (%88)
Single gland hyperplasia	17 (%7)
Parathyroid carcinoma	3 (%1)
Double adenoma	3 (%1)
Multiple gland hyperplasia	3 (%1)
No pathological gland	5 (%2)

**Figure 1:**
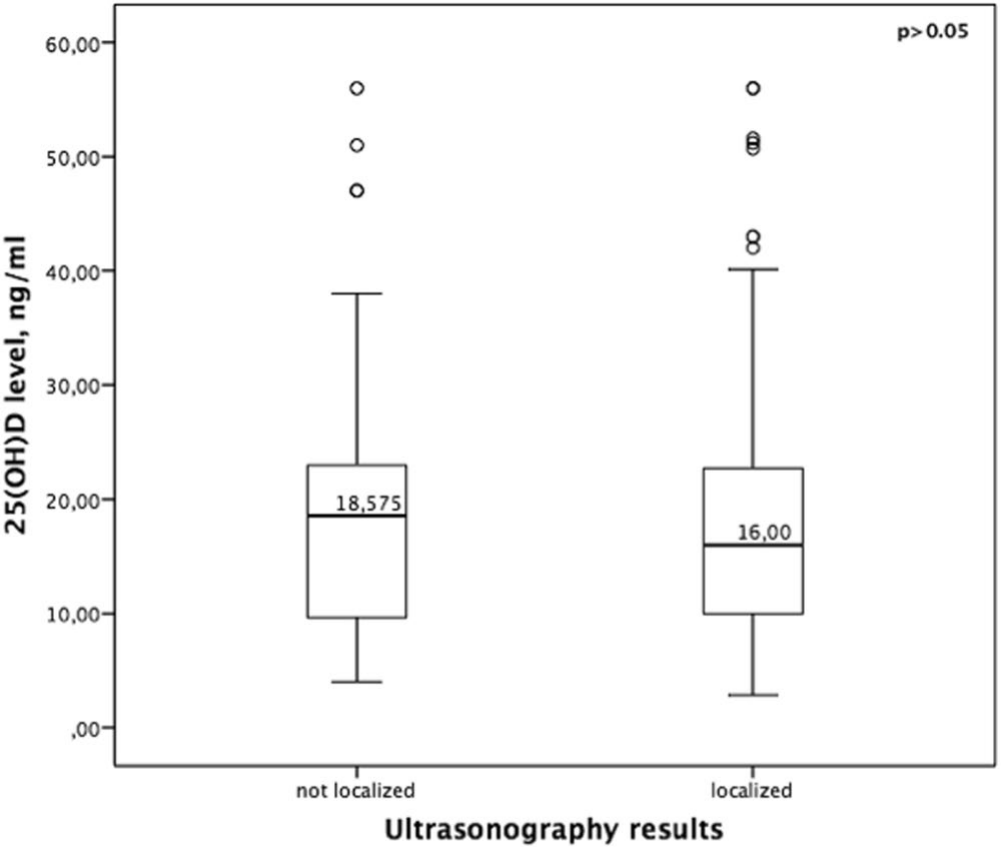
Relationship between preoperative serum levels of 25-hydroxyvitamin D (25[OH]D) and ultrasonography results.

**Figure 2:**
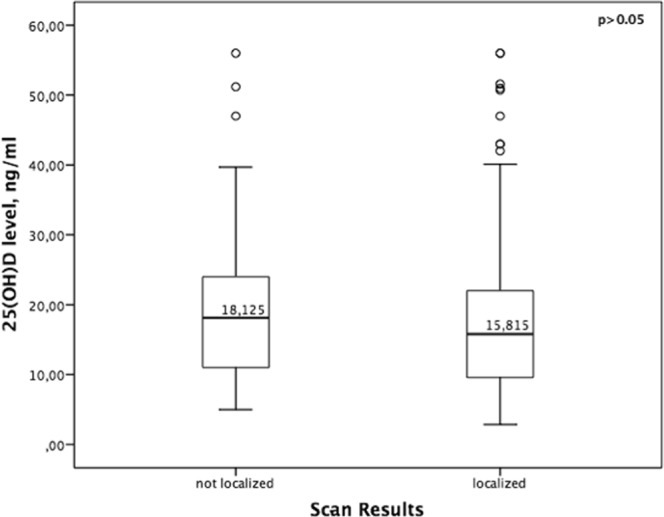
Relationship between preoperative serum levels of 25-hydroxyvitamin D (25[OH]D) and sestamibi scan results.

Sensitivity and PPV of US were %80 and %92 respectively and sensitivity and PPV of MIBI were %80.5 and %91 respectively in the whole group. No significant difference was found between the sensitivity and PPV of US and MIBI scan in the whole group (p > 0.05). US failed to localize in 52 (%20.3) patients while MIBI could not localize in 50 (%19.5) patients (Table 3). The median levels for 25(OH)D were not statistically different in patients with positive and negative US screening results (16 vs. 18.5 p > 0.05) (Fig. 1) or between patients with positive and negative MIBI screening results (15.8 vs 18.1 p > 0.05) (Fig. 2). In addition, this study showed that vitamin D level had no impact on accurate detection of adenoma by US or MIBI scan (p > 0.05). The sensitivity of US and MIBI scan decreased in vitamin D replete group but there was no significant difference in the sensitivity of US (%80.5 vs. %82 vs. %71 p > 0.05) and MIBI (%81 vs. %84 vs. %71 p > 0.05) scan between 3 groups (Table 4). The results of the multivariate logistic regression analyses showed that vitamin D level was not an independent predictor of localization on either US or MIBI scan (p > 0.05) after adjusting for different variables such as comorbid thyroid disease or Ca and PTH level. The logistic regression analyses demonstrated that higher levels of serum calcium increased the detection of abnormal parathyroid glands by US (r^2^:0.033 p:0.032) and that higher levels of serum PTH increased the detection of abnormal parathyroid glands by MIBI scan (r^2^:0.05 p:0.025). The sensitivity of US in patients without nodule was %89 and it is reduced to %76 in patients with nodular thyroid disease (p:0.017). MIBI scan sensitivity was not impaired by nodular thyroid disease (%83 to %80 p > 0.05). In patients with a history of thyroidectomy the sensitivity of both US and MIBI scan was impaired (%43 and %43 p:0.002, p:0.033 respectively).

**Table 3:** Comparison of Ultrasound and Sestamibi scan results.

Sestamibi					
	n (%)	Correctly localized	Incorrectly localized	Not localized	Total
Ultrasound	Correctly localized	178	3	23	204 (%80)
	Incorrectly localized	6	12	0	18 (%7)
	Not localized	22	5	7	34 (%13)
	Total	206 (%80.5)	20 (%8)	30 (%12)	256 (%100)

**Table 4:** Sensitivity ratios of US and MIBI.

	Whole group (%)	Vitamin D		>30 ng/dl	Dvit < 20 vs. 20–30	p value	Dvit > 30 vs. 20–30
		≤20 ng/ml	20–30 ng/ml			Dvit < 20 vs. > 30	
US sensitivity	80%	80.5%	82%	71%	0.783	0.232	0.227
MIBI sensitivity	80.5%	81%	84%	71%	0.200	0.630	0.153

US: ultrasonography; MIBI: Tc99m Sestamibi.

## Discussion

Accurate localization of parathyroid adenoma is critical for mini-invasive parathyroidectomy which has lower complication rates then bilateral neck exploration^1^. In our institution consistent with guideline recommendations, neck US and MIBI scan are used as first-line imaging, which increases the rate of successful localization of adenoma compared to a single imaging test. When there is concordance between scintigraphy and ultrasound, the PPV for successful localization of a parathyroid adenoma is very high^14^.

In this study, vitamin D deficiency and insufficiency rates were found to be %66 and %22, respectively, compatible with the literature and our country^15–17^. Although most patients diagnosed in winter, contrary to expectations, there was no significant difference in 25(OH)D levels between the four seasons. The possible explanation for this result is the increased level of PTH converts more 25(OH)D to 1,25-dihydroxyvitamin than normal individuals. Also covered dressing style and inadequate sunlight exposure in our city are effective on low 25(OH)D level in these patients. VDD is associated with larger and more active adenomas^6,7^ and this raised the question of whether vitamin D deficiency has an impact on the success of localization studies. Recently, there have been studies investigating the effect of vitamin D replacement on Ca level or PTH level in PHP patients^18–20^. However the effect of vitamin D replacement on the sensitivity of localization studies is not known. In the current study the resected adenoma volume were found to be higher in vitamin D deficient patients but no effect could be determined of vitamin D level on the sensitivity of either US or MIBI. Median vitamin D levels were found to be lower in the successfully localized patients but the difference in vitamin D level was not statistically significant. There are many studies in the literature showing that comorbid thyroid disease lowers the sensitivity of imaging tests^2,21,22^. In the current study, nodular thyroid disease prevalence was high (%66) but there was no statistically significant difference in the rate of patients with the nodular disease between the vitamin D deficient, insufficient and replete groups (p > 0.05). Furthermore, in the multivariate logistic regression analyses, vitamin D level was not an independent factor, affecting the success of the localization studies even after adjusting for different variables such as comorbid of thyroid disease or Ca and PTH level. In the vitamin D deficient subjects PTH level and resected adenoma volume were higher than in patients with sufficient vitamin D level. Therefore, although Vitamin D level is associated with disease severity or adenoma size, it was not seen to have any effect on the success of localization studies. Tassone *et al*. and Tay *et al*. also investigated the effect of Vitamin D level on localization studies and found that VDD does not affect localization studies^23,24^. However in these studies the sensitivity of the localization studies were low and one of them was missing the findings of US. Kandil *et al*. reported that MIBI scan is eight times more positive in VDD subjects, but in that study, the coexistence or distribution of NG disease was not clarified^8^.

In the current study, four factors were found to affect the success of US in the localization of adenoma; Ca level, co-existence of thyroid nodule, parathyroid adenoma volume and thyroidectomy history. Patients with previous thyroid surgery had 11.9 times more negative results on US and patients with noduler goiter had 2.7 times more negative results on US than other patients. US sensitivity was found to decrease from %89 to %76 in patients with nodular thyroid disease. Therefore MIBI scan should be the preferred localization study, especially in MNG patients. In logistic regression analysis, the Ca level was determined to be an independent predictor of successful localization in US and PTH level was an independent predictor of successful localization in MIBI scan. Sestamibi uptake is related to the activity of adenoma cells, which explains the correlation between localization accuracy and PTH level. In this study, Ca level was found to be correlated with parathyroid adenoma volume and parathyroid adenoma volume was found to be significantly higher in positive localization studies, which explains the correlation between US sensitivity and Ca level. Hughes *et al*. also showed that preoperative serum Ca and PTH levels were correlated with the sensitivity of US and MIBI scan^3^.

Previous thyroidectomy history also lowered the MIBI scan sensitivity but comorbid thyroid nodule did not change the sensitivity of MIBI Scan. Another result of this study was that adenoma weight is not related to localization studies. In the correlation analysis, the vitamin D level was negatively correlated with PTH, adenoma weight and volume, but there was no correlation detected between vitamin D level and serum Ca level this study. This is important because preoperative vitamin D replacement in PHP patients has been the subject of many recent studies^18–20^. Despite the concerns about the increase in hypercalcemia, vitamin D repletion in mild PHP patients has been shown not to result in any increase in hypercalcemia^18–20^. Therefore, vitamin D level seems to be unrelated to the degree of hypercalcemia in PHP patients similar to the result of the current study. It may also be concluded that vitamin D replacement will not affect localization studies.

In patients with parathyroid gland hyperplasia the success of imaging studies was not differ from patients with adenomas and there was also no difference in the results of US and MIBI scan in these patients. In ectopic parathyroid adenomas (%2.3) US successfully localized in %50 of patients while MIBI scan was successful in %83 of patients. Due to the low sensitivity of US in ectopic glands many clinicians choose to perform US and MIBI scan together in localization studies. However in the current study there were only six patients (%2.3) where adenoma was incorrectly localized on US and correctly on MIBI scan. All of these six patients also had nodular thyroid disease. In patients without thyroid nodules, US can be the first and only localization study. In addition, US successfully localized adenomas in %80 of patients with multi-glandular diseases, which is equal to MIBI scan success.

## Conclusions

The results of this study demonstrated that in PHP patients, vitamin D level is not related to the success of localization tests. US sensitivity significantly increased with hypercalcemia level and MIBI sensitivity increased with hyperparathyroidism level. Nodular thyroid disease was found to affect the sensitivity of US but not MIBI. There was no superiority between the sensitivities of US and MIBI scan. Except in patients with nodular goiter, US can be used as the initial and sole localization test in PHP patients.

## References

[1] Udelsman R, Lin Z, Donovan P (2011). The superiority of minimally invasive parathyroidectomy based on 1650 consecutive patients with primary hyperparathyroidism. Ann. Surg..

[2] Medas F (2016). Retrospective evaluation of the pre- and postoperative factors influencing the sensitivity of localization studies in primary hyperparathyroidism. Int. J. Surg..

[3] Hughes DT, Sorensen MJ, Miller BS, Cohen MS, Gauger PG (2014). The biochemical severity of primary hyperparathyroidism correlates with the localization accuracy of sestamibi and surgeon-performed ultrasound. J. Am. Coll. Surg..

[4] Walker MD (2015). Vitamin D in primary hyperparathyroidism: Effects on clinical, biochemical, and densitometric presentation. J. Clin. Endocrinol. Metab..

[5] Silverberg SJ (2007). Vitamin D Deficiency and Primary Hyperparathyroidism. J. Bone Miner. Res..

[6] Sudhaker Rao D (2000). Effect of vitamin D nutrition on parathyroid adenoma weight: Pathogenetic and clinical implications. J. Clin. Endocrinol. Metab..

[7] Raef H (2004). The effect of vitamin D status on the severity of bone disease and on the other features of primary hyperparathyroidism (pHPT) in a vitamin D deficient region. J. Endocrinol. Invest..

[8] Kandil E (2008). Correlation of plasma 25-hydroxyvitamin D levels with severity of primary hyperparathyroidism and likelihood of parathyroid adenoma localization on sestamibi scan. Arch. Otolaryngol. - Head Neck Surg..

[9] Darling AL (2014). Greater seasonal cycling of 25-hydroxyvitamin D is associated with increased parathyroid hormone and bone resorption. Osteoporos. Int..

[10] Moosgaard B (2005). Vitamin D status, seasonal variations, parathyroid adenoma weight and bone mineral density in primary hyperparathyroidism. Clin. Endocrinol. (Oxf)..

[11] Nevo-Shor A (2016). Seasonal changes in serum calcium, PTH and vitamin D levels in patients with primary hyperparathyroidism. Bone.

[12] Bilezikian JP (2014). Guidelines for the Management of Asymptomatic Primary Hyperparathyroidism. Parathyroids Basic Clin. Concepts Third Ed..

[13] Holick MF (2011). Evaluation, treatment, and prevention of vitamin D deficiency: An endocrine society clinical practice guideline. J. Clin. Endocrinol. Metab..

[14] Kunstman JW, Kirsch JD, Mahajan A, Udelsman R (2013). Parathyroid localization and implications for clinical management. J. Clin. Endocrinol. Metab..

[15] Samples B (2006). A very high incidence of low 25 hydroxy-vitamin D serum concentration in a French population of patients with primary hyperparathyroidism. J.Endocinol. Invest..

[16] Hekimsoy Z (2010). Vitamin D status among adults in the Aegean region of Turkey. BMC Public Health.

[17] Satman I (2013). Twelve-year trends in the prevalence and risk factors of diabetes and prediabetes in Turkish adults. Eur. J. Epidemiol..

[18] Isidro ML, Ruano B (2009). Biochemical effects of calcifediol supplementation in mild, asymptomatic, hyperparathyroidism with concomitant vitamin D deficiency. Endocrine.

[19] Tucci JR (2009). Vitamin D therapy in patients with primary hyperparathyroidism and hypovitaminosis D. Eur. J. Endocrinol..

[20] Grubbs EG (2008). Preoperative vitamin D replacement therapy in primary hyperparathyroidism: Safe and beneficial?. Surgery.

[21] Berber E (2008). Factors Contributing to Negative Parathyroid Localization: An Analysis of 1000 patients. Surgery.

[22] Erbil Y (2006). Impact of gland morphology and concomitant thyroid nodules on preoperative localization of parathyroid adenomas. Laryngoscope.

[23] Tassone F (2016). Vitamin D Deficiency Does Not Affect the Likelihood of Presurgical Localization in Asymptomatic Primary Hyperparathyroidism. Endocr. Pract..

[24] Tay YKD (2018). Pre-operative localization of abnormal parathyroid tissue by 99mTc-sestamibi in primary hyperparathyroidism using four-quadrant site analysis: an evaluation of the predictive value of vitamin D deficiency. Endocrine.

